# Atlanta Society of Medicine

**Published:** 1900-02

**Authors:** 


### SOCIETY REPORTS.

### ATLANTA SOCIETY OF MEDICINE.

Atlanta, Ga., January 18, 1900.

*Regular Meeting of the Atlanta Society of Medicine.*

Meeting was called to order by the president, Dr. J. C. Olmsted.

Dr. F. S. Bourns delivered an addressed on "Some Medical Problems in the Philippines" as follows:

When Dr. Olmsted called upon me the other day to give a paper this evening I did not feel that I could do justice to a formal paper in the short time allowed and asked to be allowed to make some informal remarks instead, in regard to some of the problems which will be presented to Americans in the Philippine Islands.

By way of preface I would like to state that for a tropical climate the Philippines have as good a one as I know of. Of course, there are many diseases found there, and probably it is easier to contract disease there than here, but I believe that with proper care as to diet and hygiene there is no reason why one should not live to a comfortable old age there as well as anywhere else. One of the great troubles with the region, they say, is the great amount of liver trouble, and they attribute this to the climate, but I rather think it is due more to the alcoholic habit than to the climate. The rate at which a foreigner in the east can dispose of brandy and soda is simply amazing and undoubtedly has a great deal to do with these liver troubles complained of. I have had an experience of almost five years in that region, and I do not think my health suffered more than in this country. It is true I had a slight attack of typhoid fever last year, but I might have had the same here.

However, there are some questions in the Philippines which will have to be taken up by the American authorities. Spanish rule was about one hundred years behind the times, and this also applied to medical matters to a certain extent. They were not so far behind in regard to quarantine but were particularly so as to sanitary matters. The sanitary conditions in Manila were the poorest when we went there. Manila is a sort of Venice and is cut through by many canals, the tide falling six feet once or twice a day. The tide is quite an aid in draining the city, as in falling it produces a strong current in the river and canals, and in this way a great deal of the sewage is carried off.

I will not speak of the work we had to do to get things in reasonable shape. It was very hard work indeed. The greatest thing we had to deal with was an army of 13,000 Spanish prisoners who did not know the first thing about sanitary matters. We could not even get them to clean up their quarters. We offered them all disinfecting material for their use, but they would not take it. One doctor did come to me to get some chloride of lime, and when I asked him how much he wanted he stated that if I could spare it he would like three or four pounds. I gave him fifty pounds and ten gallons of carbolic acid and told him he could get more when that gave out. The amount I gave him was sufficient to last four or five days for the purpose for which he desired it. He came back at the end of a month and wanted some more. This gives you some idea of the way in which they carry on disinfection.

I want to speak particularly of some of the diseases in Manila and the islands and where the most work will have to be done. I think the two most important diseases are smallpox and leprosy.

Smallpox in the Philippine Islands is an endemic disease. I have frequently seen children with the eruption out playing in the streets of the towns in the various islands of the group. The people themselves have no fear of the disease, as they are fatalists in regard to it. Although they are Christians in the modern sense of the word, they are very superstitious. They believe that if a person is to die with the disease they will die and nothing can stop it. You will therefore find a child with smallpox and other children playing around it. Also, if a child is carried off by the disease, they will have a funeral feast that night and all the neighbors will come to it. The Filipinos are very fond of eating, and never neglect an opportunity to do so, but go to a feast whenever they can and this is one of the occasions. This, you see, favors the spread of the disease, and it is found endemic throughout the year, as you can always find a few cases. In certain months there is almost certain to be a decided increase in the disease. This applies to the provinces as well as to Manila. According to the reports for 1895 and '96. I found that during March, April, and May the number of deaths from smallpox in Manila was about 250 each month, and that was not considered an epidemic but only a slight increase. Vaccination was supposed to be compulsory. It was in theory but not in practice, and this was due to the corrupt methods of the Spaniards. They had a place to manufacture vaccine, but how much in all was really made I do not know. They had scattered about the country public vaccinators. These were paid very small salaries, and they collected the balance from the people, and the way they did their work was very poor indeed, as I found a large number in Manila not vaccinated, in children the proportion being very small. Very frequently the vaccinators in the provinces, being out of vaccine, would go about scarifying the arms of the children and rubbing in a little molasses. They would go through the form simply to get their fees, and this accounts for the disease being endemic and carrying off so many of the children. We looked at this as something to be overcome. We first encountered smallpox in November of 1898, it breaking out simultaneously in the Spanish army and American army. We proceeded immediately to vaccinate. I collected a few facts in regard to the results of our vaccinations in the way of reducing smallpox. The surgeon in the American army was much rushed with work and asked me as Health Officer of Manila to vaccinate the Spanish soldiers who were in the city. I finally received instructions from headquarters to do this, and we started a vaccine institute, using the water buffalo, as I found this a very good animal for the production of vaccine, and I preferred it to the native cattle as I had been assured that it was not subject to tuberculosis. I was told that it was not necessary to vaccinate these Spaniards, as it had been done before they came into that country. I replied that I would vaccinate them anyway. In two weeks time there had been seven cases of smallpox in the Spanish army. We vaccinated 6,000 of these and I received reports on 2,000, and these reports showed 82 per cent. of successful vaccinations. This may have been a little high, as they may have reported some pseudo-reactions as being true reactions. At any rate, from that time on we did not have a case of smallpox in the Spanish army. I think this speaks strongly in favor of vaccination.

We had comparatively little smallpox in the city. I vaccinated as fast as means were given me. I had some delay in obtaining the necessary means, and I urged upon the authorities to vaccinate before we had an outbreak of the disease. The outbreak occurred in February, and during that time had the most favorable conditions for an epidemic. This was due to the burning of the suburbs around Manila, the whole population crowding into the center of the city, the density of population being increased about 100 per cent. The result was seen in about ten or twelve days, that is, about the period of incubation of smallpox. We had a smallpox hospital in which there were about ten cases, and in one week these were increased to 100, and we saw that we had to deal with an epidemic. The authorities recognized this and immediately placed unlimited funds at my disposal to control the disease and stamp it out. We had vaccinated 20,000 children up to that time, and we then vaccinated about 60,000 in the next few weeks. This stamped out the disease, and we had no cases in the city after that.

This plan of vaccination and isolation, if adopted throughout the provinces, will make it the disease as found in this country. The natives were at first strongly opposed to isolation and such precautions, and I sometimes had to send a guard to the house to convince the people that they must be vaccinated. But this was in time overcome when they saw the value of it and found that they would receive better treatment at the hospital, and after this 50 per cent. of those in the hospital presented themselves voluntarily. The natives are different from our negro here, as they are very intelligent and quick to recognize the value of new methods. I say this to show that they take up new ideas as soon as they see they are good ideas and are the best for them.

The next problem of importance is leprosy and this is more difficult to deal with. There are two leper hospitals in the islands; one at Manila and the other at Cebu. The one at Manila can accommodate 200 patients, and during the siege of Manila these were let loose and became scattered throughout the city, and after the capture of Manila we had some trouble in finding all of these and getting them back to the hospital. This was especially the case when they were among the Chinese. The hospital at Cebu can accomodate 100 lepers but there are about 500 in the island. As to the total number of lepers in the islands, I have heard the estimate put as high as 5,000, but I think there are not over 2,500 at this time. There is very little isolation, as they are allowed to go about and therefore the disease is very common. Measures will have to be adopted toward isolation. I think the plan adopted in the Hawaiian Islands is probably the best. There are a number of small islands in the Philippine group, and one of these islands could be set aside and a colony established there. Fortunately leprosy is very rarely seen among the Europeans, but I cannot say the same thing about smallpox. These lepers are scattered from the most northern part of Luzon to the southern part of Mendanao. They can be seen about Manila occasionally, and at Cebu they can be seen almost at any time, in groups, begging on the streets.

These are important problems, but there are still others, such as improvement of the sanitary condition of Manila. This can be done only by the expenditure of a large amount of money. The land is very low, but at the same time, they have the aid of the canals and tides.

Malarial diseases are very common throughout the islands. In some regions we find the pernicious forms and in others the ordinary forms. The history of malaria there is about the same as in this country. After the land is cleared up and cultivated it will disappear. The people recognize the fact that it is a dangerous proceeding to break up new land, and therefore it is sometimes difficult to get laborers for this. They also recognize that certain regions are more malarial than others. This is especially the case with the island of Mindoro, which is just south of Luzon. When I was in the islands before, Professor Worcester and I decided to visit a place in Panay, and we were deserted by our servants, as they said there were demons in that region. We laughed at them, and visited the place, where we staid ten days, and on our return my friend was taken down with pernicious malarial fever. The demons in that region were the protozoa of malaria. The natives tell a story of a party of twelve Englishmen who visited a dangerous region, and four or five of them died while there. Of course these conditions can be improved by modern methods of sanitation. This has been shown by an energetic Spanish official in one region, where he almost eradicated the disease.

Bowel troubles are about the same as in any tropical region. They cause more sickness in the army than anything else. Here diet and hygiene play an important part. During the four years of residence in the islands I was not troubled with this, and the troubles in the army were very largely accounted for by the indiscretions on the part of the men. The men had not been paid off for two months previous to their arrival at Manila; they were paid off when they reached there, and they spent their money in every way possible, eating and drinking everything they came across, and I think this was largely accountable for the dysenteric troubles, as those who were careful were not troubled while in this way.

Typhoid fever is not very common, that is, not as common as it was in the army camps in this country. You will occasionally see a case, but never at all like the epidemics seen here. We were very fortunate in not having any epidemics in the camps such as were seen at Chickamauga and other places. The Philippines are entirely free from certain diseases. Yellow fever is not known there, as it has never been introduced and is not an endemic disease. Cholera is not seen as an endemic disease, but becomes epidemic when introduced from India and China. The last epidemic of cholera was in 1882, when, in the province of Zamboanga 36 per cent. of the population was attacked, and 50 per cent. of these died—that is, 18 per cent. of the population was swept away. As the disease is not endemic it should be kept out of the islands by strict quarantine regulations.

The plague was never seen in these islands before the present time. This was due to the Spanish being careful in regard to quarantining vessels from infected ports. The disease has prevailed in Hong Kong for the past three or four years, and when I was at Manila we were on the watch to prevent it reaching that city. I see from the papers that some cases have now been found there, and the conditions for the spread of the disease are very favorable in spite of the sanitary precautions that are being taken. The conditions for the disease are especially favorable among the Chinese, where you will find ten men in a place where three ought to be; and as they are very secretive in their nature, it will not be easy to find a case should it occur among them. The natives are also very much crowded together at this time, and the ordinary vermin which can convey the disease, such as rats, etc., are very common. Also, at the present time there is more or less distress in the city on account of the high prices of foodstuffs, as there is from 25 to 100 per cent. advance over normal prices. This entails some privations on certain elements in the city, making the conditions more favorable for an epidemic than at normal times. Therefore, unless vigorous measures are taken, the disease may gain a foothold in the city. We had an illustration in May of last year of the favorable soil in an epidemic of beriberi, which is endemic on the islands. Fortunately this is a disease which does not attack Europeans, but is fatal among the natives, the death-rate running up as high as 25 per cent. I saw a statement in the American papers that beriberi had broken out because embalmed beef was fed to the natives. Of course this was ridiculous, as the disease was found outside the city in other districts. In regard to beef itself, I may say the Filipinos never had anything so good as that, and they were happy to get it.

Frambesia, or yaws, is not found in the Philippines, and it is very fortunate that it is absent. It is very common in the Island of Borneo, where many small children have it, and it is very fatal with them.

Most of the other diseases, such as scarlet fever, measles, whooping-cough, are not so very common. Venereal diseases are very common in the cities, but not many cases are found out in the country. Tuberculosis is probably as prevalent there as it is here, and the number of deaths from it is quite large, being about the same ratio as in this country. It is seen in all forms, the pulmonary form being most common.

Taking all things into consideration, I do not see any great difficulty in dealing with the medical problems in the Philippines. They simply need the methods we are using in this country, a little energy and the prompt use of money. If these measures are carried out, in a few years the Philippines will be made the most healthful of tropical countries. The objection is raised that it is a very hot country. This is exaggerated, as the temperature runs from 70 to 95 degrees, and is usually in the 80's. One does not suffer as much from the heat there as in Atlanta, Chicago, New York, or other large cities, and the reason for it is, that there they dress for the climate and here we do not. We continually wear heavy clothing in this country on account of the sudden changes of weather, and there they do not have any sudden changes of temperature, but have a more even temperature, and therefore can dress comfortably. I have never suffered as much from the heat in Manila as I have in some of the cities of this country. June, July and August are the hottest months of the year. About 140 miles north of Manila there are some highlands that have a temperature ranging from 32 to 70 degrees. This is a region where, by spending a small amount of money, a delightful summer resort can be established, to which foreigners can go during the summer months.

---

THE daily papers of Boston, under large head-lines which he who runs may read, advertise "Absent Treatment" with "Ninety-seven per cent. of cases successful," using an editorial from the *Medical Brief* in support of its methods. It is not necessary to say that the whole scheme is most quackishly put together and displayed, with a view to catching the unwary, and from the list of victims we should judge it had caught a goodly number. People like to be humbugged and there is plenty of opportunity for them to be satisfied. New York is just now being treated by a magician, who claims to cure deafness by some wonderful power which savors of this "absent treatment."—*Medical Times.*

